# Functional Connectivity Predicting Transdiagnostic Treatment Outcomes in Internalizing Psychopathologies

**DOI:** 10.1001/jamanetworkopen.2025.30008

**Published:** 2025-09-03

**Authors:** Kai Zhang, Heide Klumpp, Jagan Jimmy, K. Luan Phan, Mohammed R. Milad, Zhenfu Wen

**Affiliations:** 1 Faillace Department of Psychiatry and Behavioral Sciences, McGovern Medical School, University of Texas Health Science Center at Houston, Houston; 2 Department of Psychiatry, University of Illinois Chicago, Chicago; 3 Center of Innovation for Complex Chronic Healthcare, Edward Hines, Jr Veterans Administration Hospital, Hines, Illinois; 4 Department of Psychiatry and Behavioral Health, The Ohio State University, Columbus

## Abstract

**Importance:**

Predicting treatment outcomes for internalizing psychopathologies (IPs), such as depression and anxiety, holds promise for advancing precision medicine. The extent to which whole-brain functional connectivity (FC) can predict treatment responses for patients with IPs across different therapeutic modalities remains unclear.

**Objective:**

To examine whether pretreatment FC patterns predict multidimensional treatment outcomes in patients with IPs and whether predictive performance generalizes across diagnoses and treatment modalities.

**Design, Setting, and Participants:**

This prognostic study analyzed baseline neuroimaging and clinical data from patients with IPs enrolled in 1 of 2 randomized clinical trials (conducted from December 2013 to February 2018 and September 2017 to December 2020). Data analysis for predictive modeling was conducted from September 2024 through March 2025.

**Exposures:**

Participants were randomized to receive 12 weeks of cognitive-behavioral therapy (CBT), selective-serotonin reuptake inhibitor (SSRI) treatment, or supportive therapy (ST).

**Main Outcomes and Measures:**

A regularized canonical correlation analysis model was trained with pretreatment FC patterns. The ability of the model to predict multidimensional treatment outcomes spanning depression, anxiety, worry, rumination, and emotion regulation was tested. The predictive model was evaluated across diagnostic categories and treatment modalities.

**Results:**

In 181 patients with IPs (mean [SD] age, 27.7 [9.2] years; 127 women [71%] and 52 men [29%]) randomized to receive CBT (n = 89), SSRI treatment (n = 46), or ST (n = 46), baseline whole-brain connectivity robustly predicted multidimensional symptom changes. Predictions were significant at the individual level (*r* = 0.37, *P* = .009, permutation test), across diagnoses (*r* = 0.24, *P* = .02) and across treatment modalities (ST: *r* = 0.28, *P* = .02; SSRI treatment: *r* = 0.39, *P* = .006; and CBT: *r* = 0.32, *P* = .003). Connections significantly contributing to the FC variate were distributed across the brain, but especially within the default mode network and the dorsal and ventral attention networks. Predictive performance decreased in models incorporating fewer neural systems or clinical outcome dimensions.

**Conclusions and Relevance:**

In this prognostic study assessing predictive models of 181 patients with IPs, whole-brain FC reliably predicted multidimensional treatment outcomes across diagnoses and treatment modalities. These results suggest an association between neural connectivity patterns within specific neural networks and clinical improvements induced by varying treatment modalities, thereby advancing efforts toward personalized treatment approaches in psychiatry.

## Key Points

**Question:**

Can treatment outcomes for patients with internalizing psychopathologies be predicted using whole-brain functional connectivity patterns across different diagnoses and therapeutic modalities?

**Findings:**

In this prognostic study of predictive models including 181 patients with internalizing psychopathologies randomized to receive 12 weeks of cognitive behavioral therapy, selective serotonin reuptake inhibitor therapy, or support therapy, whole-brain functional connectivity significantly predicted multidimensional symptom changes across individuals, diagnostic categories, and treatment modalities.

**Meaning:**

These findings suggest that functional connectivity patterns may serve as transdiagnostic neural markers of treatment responses across treatment modalities, highlighting the potential of connectome-based predictive models to guide personalized treatment strategies in psychiatry.

## Teaser

This prognostic study uses predictive modeling to investigate whether pretreatment whole-brain functional connectivity patterns can be used to predict multidimensional treatment outcomes for patients with internalizing psychopathologies and whether predictive performance generalizes across diagnoses and treatment modalities.

## Introduction

Internalizing psychopathologies (IPs), including depression and anxiety, are among the most prevalent, costly, and disabling psychiatric conditions.^1,2,3^ Despite advances in treatment, individual responses to first-line treatments vary widely, ranging from 38% to 87% across studies,^4,5^ with lower efficacy in cases of comorbid psychopathology.^6^ Identifying neurobiological markers that can capture heterogeneity to treatment outcomes and that may inform a clinician about optimal treatment options for a given patient is critical for a neurobiological understanding of treatment variability and for developing more accurate and personalized treatment strategies.

A major challenge in predicting treatment outcomes for IPs is the transdiagnostic nature of these disorders. Depression, anxiety, and related conditions frequently co-occur, and symptom dimensions often cut across traditional diagnostic categories.^7^ This transdiagnostic overlap contributes to heterogeneity in treatment response across different symptom dimensions, hindering the performances of predictive models. Recent studies suggest that neuroimaging tools, such as functional magnetic resonance imaging (fMRI), may provide objective markers of treatment response in IPs.^8,9,10,11,12,13^ For example, pretreatment activity in the prefrontal cortex and anterior cingulate cortex are associated with symptom improvements following cognitive behavioral therapy (CBT) or treatment with selective serotonin reuptake inhibitors (SSRIs) across various tasks in patients with IPs.^14,15,16^ Using machine learning algorithms, previous studies have shown that treatment outcomes of CBT for patients with anxiety disorders can be predicted by baseline functional connectivity (FC) patterns.^17^

While connectome-based machine learning models have demonstrated promising results in predicting treatment outcomes, several challenges exist. First, the reliability of predictive models to date may be limited. Most studies included fewer than 50 participants, which raises concerns about model overfitting due to small sample sizes.^12,18^ Second, it remains unclear whether these models are generalizable across diagnoses and treatment modalities. Most predictive models are developed within a single diagnostic category and for a specific treatment modality,^11,19,20,21^ for example, CBT for social anxiety disorder. Third, existing predictive frameworks often rely on the prediction of a single clinical outcome measure, thus raising a concern about the inability to capture the multidimensional nature of symptom changes and neurophysiological adaption involved in the treatment procedure.^22,23^

These challenges highlight the need for a predictive framework that integrates transdiagnostic and multidimensional features to improve model generalizability and neurobiological interpretability. In this study, we proposed a transdiagnostic-therapeutic predictive framework using a data-driven approach—regularized canonical correlation analysis^24^ (rCCA)—to link whole-brain FC patterns with multidimensional treatment outcomes (eFigure 1 in Supplement 1). We leveraged a relatively large sample (N = 181) of patients diagnosed with major depressive disorder, social anxiety disorder, generalized anxiety disorder, panic disorder, posttraumatic stress disorder, or persistent depressive disorder. Participants underwent multiparadigm fMRI scans at baseline and were randomized to receive a 12-week course of SSRI treatment, CBT, or support therapy (ST). We used cross-diagnosis, cross-treatment modality and cross-individuals validation procedures to test the performances of baseline FC patterns in predicting clinical metrics spanning depression, anxiety, worry, rumination, and emotion regulation. Our study goal was to provide a novel transdiagnostic and transtherapeutic framework to identify connectivity biomarkers predictive of multidimensional treatment outcomes in IPs.

## Methods

This prognostic study was designed and reported in accordance with the Transparent Reporting of a Multivariable Prediction Model for Individual Prognosis or Diagnosis (TRIPOD) reporting guideline. Data were collected from December 2013 to February 2018 (ClinicalTrials.gov Identifier, NCT01903447)^25^ and from September 2017 to December 2020 (ClinicalTrials.gov Identifier, NCT03175068),^26^ and data were analyzed from September 2024 to March 2025. All procedures were approved by the University of Illinois at Chicago institutional review board, and written informed consent was obtained from all participants. Participants were compensated $15 per hour for their time, and all procedures complied with the Declaration of Helsinki.^27^

### Participants

Data were obtained from 2 previously conducted clinical trials (ClinicalTrials.gov Identifiers, NCT01903447 [clinical trial 1] and NCT03175068 [clinical trial 2]). Only patients who completed the baseline fMRI scans and the full 12-week treatment cycle, including assessments before and after treatment with complete clinical data, were included in the analyses (N = 181). Clinical trial 1 included participants (N = 87) with a range of IPs, including major depressive disorder, social anxiety disorder, generalized anxiety disorder, panic disorder, posttraumatic stress disorder, and persistent depressive disorder, who were randomly assigned to receive a 12-week course of SSRI (n = 46) or CBT (n = 41). Clinical trial 2 included participants (N = 94) with social anxiety disorder or major depressive disorder diagnosis that were randomly assigned to a 12-week course of ST (n = 46) or CBT (n = 48). Details about exclusion and inclusion criteria are provided in the eMethods in Supplement 1.

### Treatment Procedures

Data collection, all of which was conducted at the University of Illinois at Chicago, ceased on reaching the predetermined sample size. Participants were randomized to treatment arms through randomization using a random number generator, and assignments were made by research coordinators. Participants were randomized to the SSRI or CBT arm in clinical trial 1, and in clinical trial 2, they were randomized to either CBT or ST arms. All participants were free of pharmacotherapy and psychotherapy at baseline. Details for treatment procedures can be found in the eMethods in Supplement 1.

### Evaluation for Treatment Outcome

Treatment response was assessed using 8 clinical metrics: Beck Depression Inventory, Hamilton Rating Scale for Anxiety, Hamilton Rating Scale for Depression, Penn State Worry Questionnaire, Liebowitz Social Anxiety Scale, Ruminative Response Scale, Emotion Regulation Questionnaire (suppression and reappraisal subscales). These metrics were assessed at baseline and posttreatment. Treatment outcome was defined as the change from before to after treatment (difference scores) collapsed across metrics.

### Neuroimaging Data Acquisition and Processing

Neuroimaging data were acquired using a 3T GE scanner and preprocessed with fMRIPrep version 20.0.2.^28^ The FC matrix was constructed using a 442-region whole-brain atlas comprising 400 cortical regions,^29^ 32 subcortical regions,^30^ and 10 cerebellar regions.^31^ More details are provided in the eMethods in Supplement 1.

### Predictive Modeling Based on rCCA

We used rCCA to predict treatment outcomes from FC. The rCCA model identifies maximally correlated components between FC measures and clinical metrics, with L2 regularization to reduce overfitting and improve stability in high-dimensional data. To further mitigate overfitting, principal component analysis was conducted to reduce dimensionality of FC measures before use in the rCCA. A grid search procedure was performed to identify the optimal combination of the regularization parameter (ranging from 0 to 1 with increments of 0.05) and the number of principal component analysis components (selected from a candidate range in increments of 5 to reduce computational load, but comparable results could be obtained with finer increments, ie, step size of 1) (eFigure 2 in Supplement 1). To avoid overfitting and double dipping, the grid search was conducted using an inner 5-fold cross-validation procedure based only on the training set, and the selected parameters were directly applied to the test set. Although the rCCA yields multiple canonical variate pairs, we focused on the first pair, which captured the highest brain-symptom covariance and demonstrated statistically significant performance based on a permutation test procedure. (eFigure 3 in Supplement 1).

### Validation Strategies

To assess the generalizability of the predictive model, we implemented 3 different cross-validation strategies: standard *k*-fold cross-validation, cross-diagnosis validation, and cross-treatment modalities validation. These strategies aimed, respectively, to test generalization across new individuals, unseen diagnostic groups, and different treatment modalities (eMethods in Supplement 1).

### Prediction Using a Subset of Networks and Clinical Metrics

In the main analysis, we used whole-brain FC patterns to predict the treatment outcomes. This approach was informed by emerging evidence suggesting that distributed neural systems are integrated for diverse cognitive and emotional functions,^32,33,34^ and thus may better capture individual variability in treatment response compared with localized neural patterns. To examine this point, we conducted additional post hoc analyses by restricting the predictive model to connections within specific networks or between subsets of networks. Additionally, we examined the impact of using a single vs multiple clinical metrics to assess the effect of clinical metric dimensionality on predictive accuracy.

### Statistical Analysis

To assess the statistical significance of our predictive models, we conducted a nonparametric permutation test by randomly shuffling the clinical metrics across participants, thereby disrupting the association between FC patterns and symptom measures. We then performed the same aforementioned cross-validation procedures and generated a null correlation distribution across 1000 permutations of the input data. The *P* value was computed as the proportion of null correlations that exceeded the correlation score obtained from the nonpermuted data.

For the identification of FC features that significantly contributed to the predictive models, we retained the canonical variables corresponding to the FC matrix from the rCCA computation. Significant connections were identified by calculating the correlation between the canonical variate and all connectivity edges, followed by false discovery rate correction (2-sided *P* < .05) to control for multiple comparisons. In addition, a nonparametric bootstrap procedure (1000 iterations) was used to assess the repeatability and stability of significant connections and clinical-scale loadings. All analyses were conducted with Python, version 3.11.6 (Python Software Foundation), using Nilearn, version 0.11.1 (Inria Mind), scikit-learn, version 1.6.1 (Inria), and CCA-Zoo, version 2.6.0 (CCA-Zoo).

## Results

### Prediction of Treatment Response Across Individuals, Diagnoses, and Treatment Modalities

**Figure 1.**
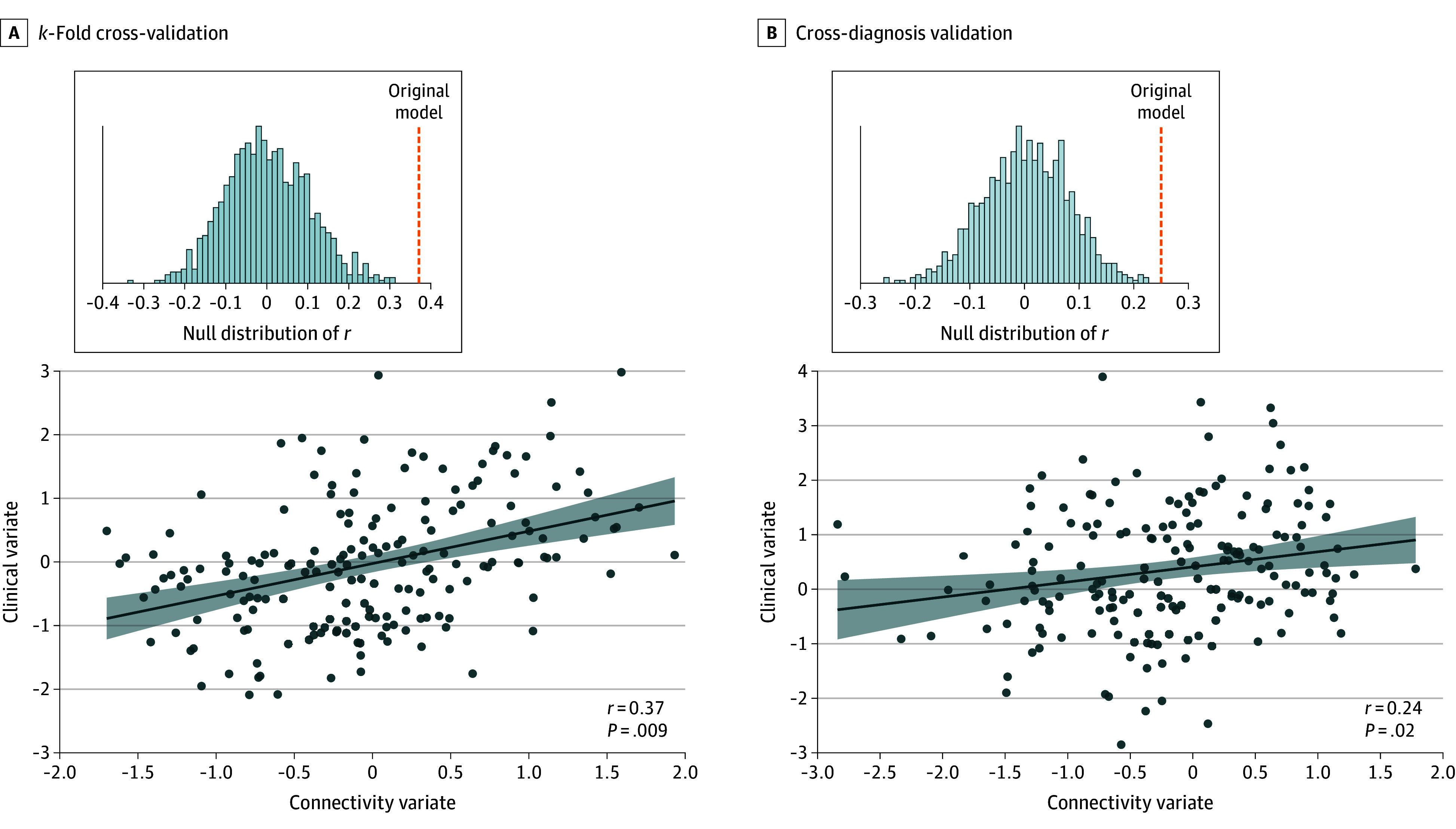
Prediction of Multidimensional Treatment Responses A, Performance based on 10-fold cross-validation. B, Performance based on cross-diagnosis validation. Solid circles represent individual participants; the solid line, linear regression fit; and the shaded area, 95% CI of the fit.

A total of 181 participants (mean [SD] age, 27.7 [9.2] years; 127 women [71%] and 52 men [29%]) were included in the predictive analyses. The rCCA model identified 1 generalizable pair of canonical variates associated with brain connectivity and treatment measures. Specifically, a 10-fold standard cross-validation procedure indicated that the pretreatment FC measures significantly predicted treatment outcomes across individuals (*r* = 0.37, *P* = .009) (Figure 1A). In cross-diagnosis validation, in which 1 diagnosis group was left out as testing set, the prediction performance remained significant (*r* = 0.24, *P* = .02) (Figure 1B). Significant prediction could also be replicated when using the percentage of symptom change as the treatment outcome (eFigure 4 in Supplement 1).

**Figure 2.**
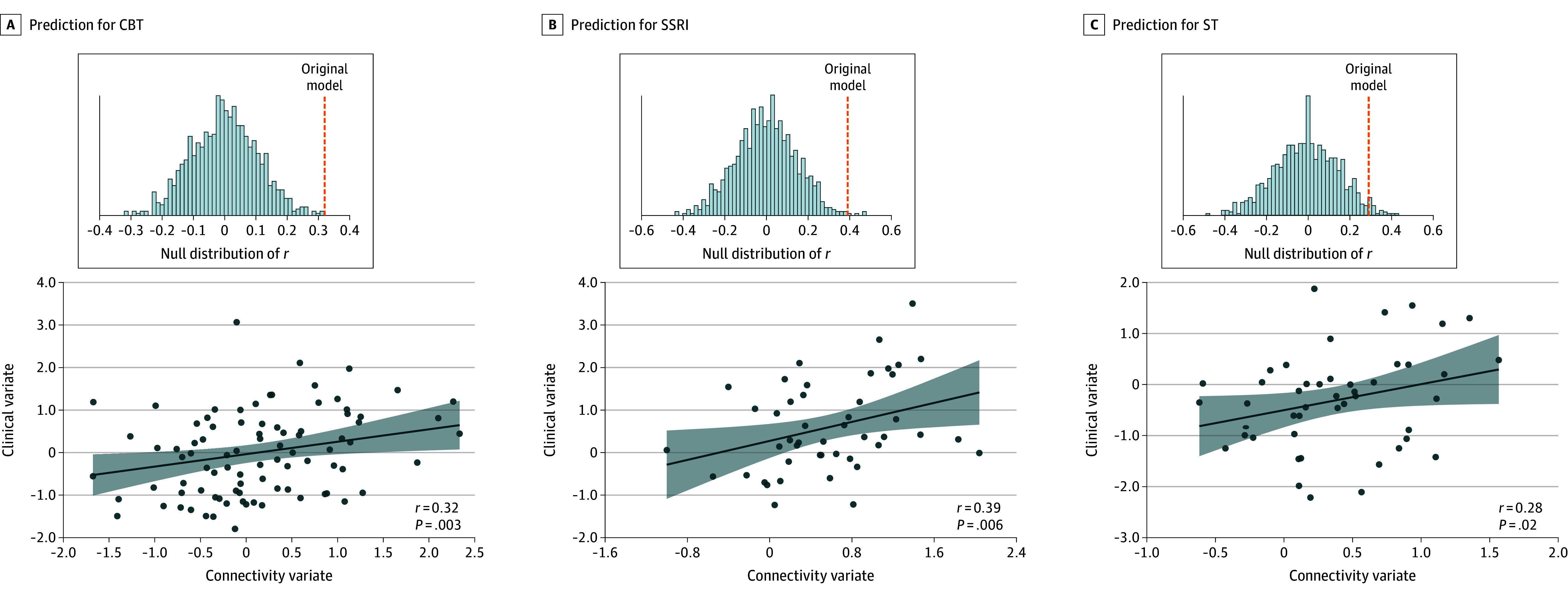
Prediction Performance Under Cross-Treatment Modalities Validation CBT represents cognitive behavioral therapy; SSRI, selective serotonin reuptake inhibitor; and ST, supportive therapy. Solid circles represent individual participants; the solid line, linear regression fit; and the shaded area, 95% CI of the fit.

In cross-treatment modalities validation, the model trained on 2 treatment modalities significantly predicted outcomes in the third modality (Figure 2). The best performance was observed in the SSRI group (*r* = 0.39; *P* = .006), followed by CBT (*r* = 0.32; *P* = .003) and ST (*r* = 0.28; *P* = .02) groups.

### Features Contributing to the Canonical Variates

**Figure 3.**
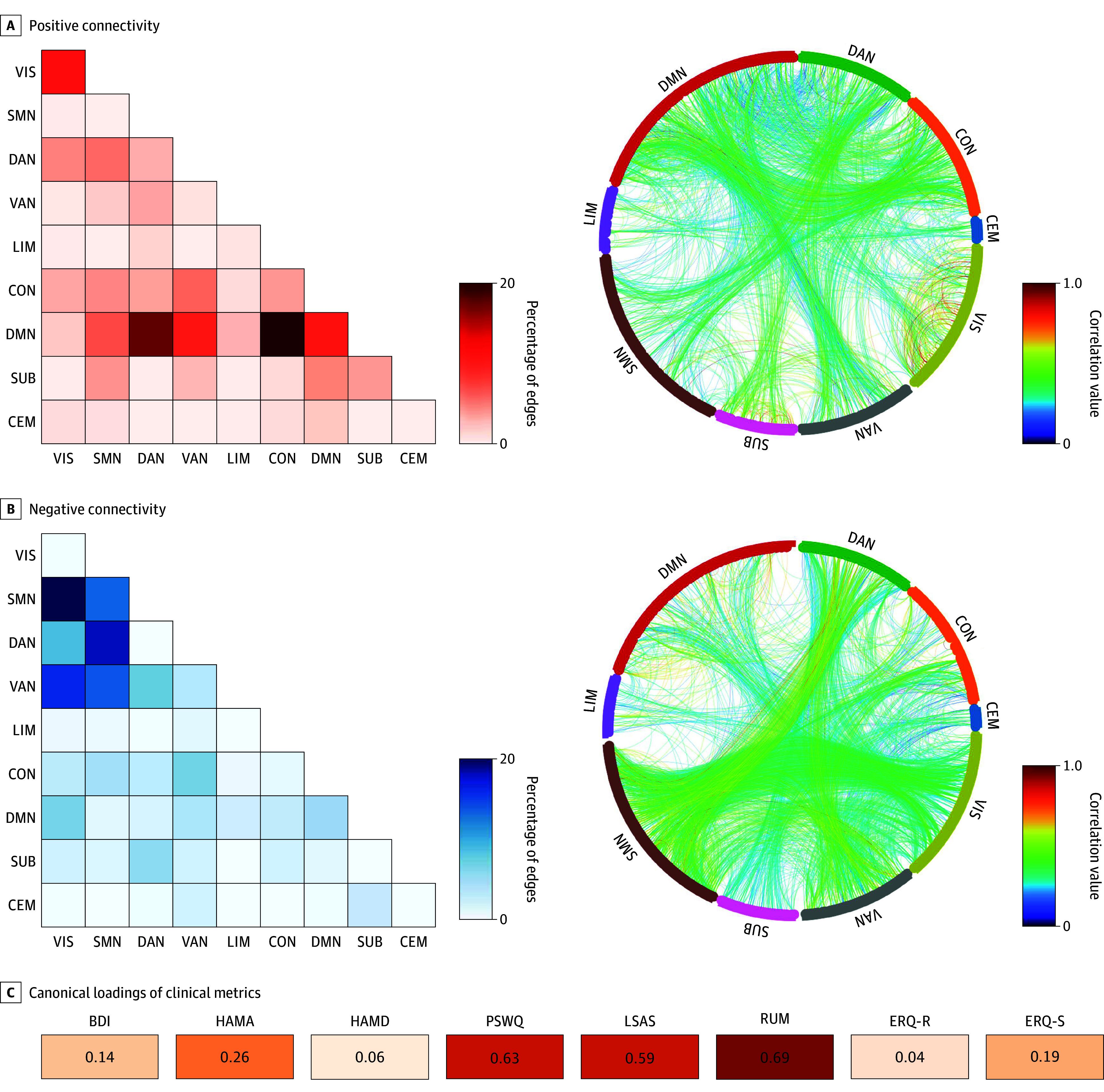
Features Contributing to Canonical Variates A, The proportion of edges (left) and chord diagrams for edges (right) positively and significantly contribute to the prediction within or between the 9 subnetworks. B, The proportion of edges (left) and chord diagrams for edges (right) negatively and significantly contribute to the prediction within or between the 9 subnetworks. The color assignment is derived from the normalized regularized canonical correlation analysis weights corresponding to each edge. C, The canonical loadings of the clinical metrics represent the contribution of each metric to the prediction. BDI represents Beck Depression Inventory; CEM, cerebellum network; CON, frontoparietal control network; DAN, dorsal attention network; DMN, default mode network; ERQ-R, Emotion Regulation Questionnaire Reappraisal; ERQ-S, Emotion Regulation Questionnaire Suppression; HAMA, Hamilton Rating Scale for Anxiety; HAMD, Hamilton Rating Scale for Depression; LIM, limbic network; LSAS, Liebowitz Social Anxiety Scale; PSWQ, Penn State Worry Questionnaire; RUM, Ruminative Response Scale; SMN, sensorimotor network; SUB, subcortical network; VAN, ventral attention network; VIS, visual network.

We then examined how the FC features and clinical measures contributed to the significant canonical variates identified by the predictive model. This analysis revealed 4875 FC edges that significantly contributed to the FC variate (*P* < .05 for false discovery rate). Of these, 2122 edges (44%) showed a positive correlation with the FC variate (Figure 3A), predominantly involving connections between the default mode network (DMN) and other networks, including the dorsal attention network (DAN), ventral attention network (VAN), and frontoparietal control network (CON). In contrast, 2753 edges (56%) exhibited a negative correlation with the FC variate (Figure 3B). These edges were primarily distributed within and between the sensorimotor network (SMN), VAN, DAN, and visual network (VIS).

The Ruminative Response Scale, Penn State Worry Questionnaire, and Liebowitz Social Anxiety Scale were the top-loading scales on the symptom side canonical variate (Figure 3C). Consistent with their dominant influence, an exploratory analysis showed that patients who achieved top-tier improvement on these same 3 scales exhibited significantly higher predicted canonical symptom scores than did lower-tier responders (eFigure 6 in Supplement 1), underscoring the clinical relevance of the derived variate.

A nonparametric bootstrap procedure (1000 iterations) replicated the contribution patterns on the brain side and the symptom side. This result demonstrated the stability of both canonical variates (eFigure 5 in Supplement 1).

### Prediction Using a Subset of Networks and Clinical Metrics

**Figure 4.**
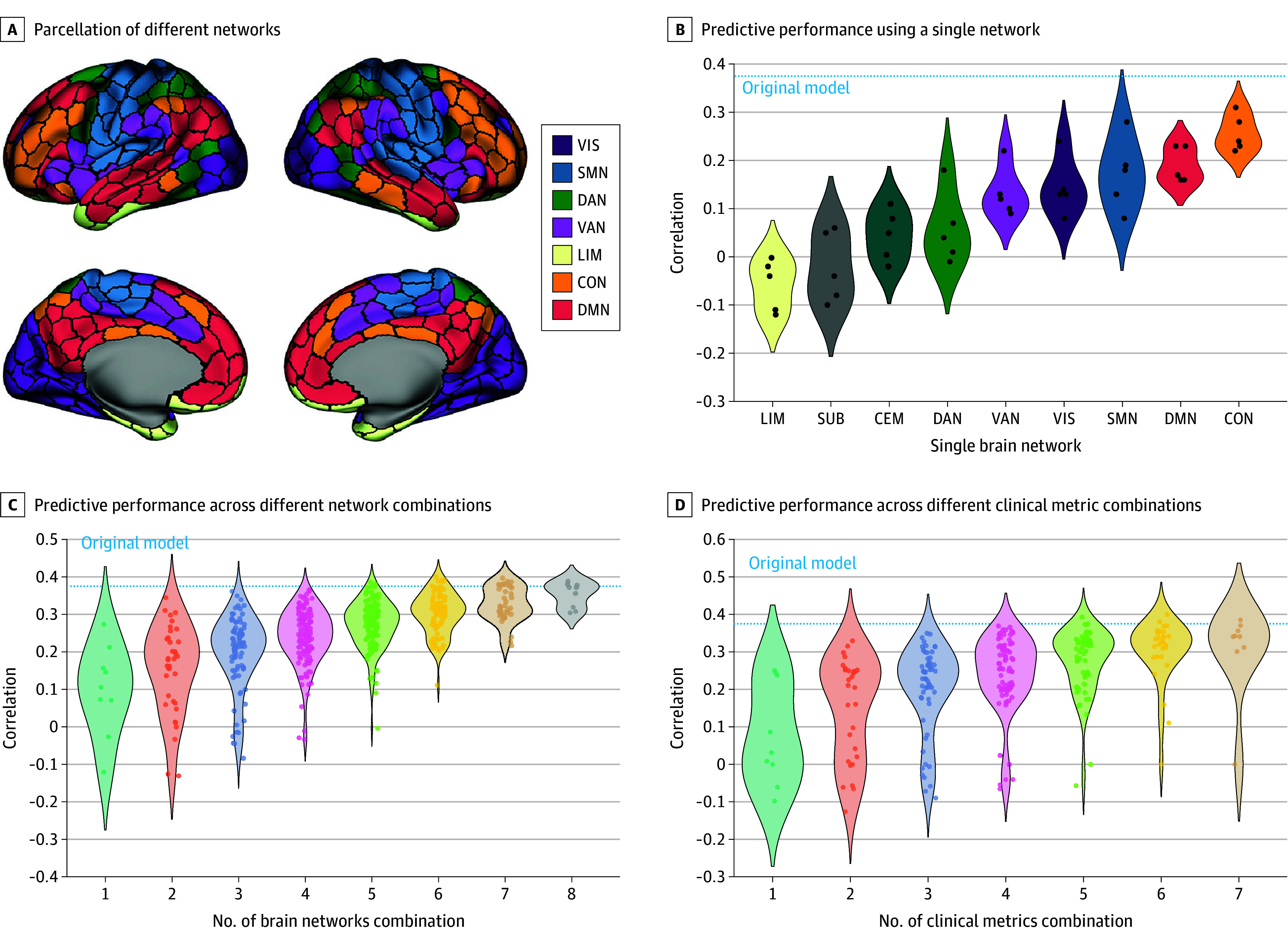
Prediction Performance Using a Subset of Networks and Clinical Metrics A, Parcellation of different networks based on the Schaefer 2018 Yeo-7 network.^29^ Analysis used the 7 networks plus the subcortical and cerebellar networks. B, Each dot within a violin represents an *r* value from a different random seed for participants splitting during 10-fold cross-validation. C, Each dot within a violin represents an *r* value from a specific network combination, with the number of networks used indicated on the x-axis. D, Each dot with a violin represents an *r* value from a specific metric combination, with the number of metrics used indicated on the x-axis. CEM represents cerebellum network; CON, frontoparietal control network; DAN, dorsal attention network; DMN, default mode network; LIM, limbic network; SMN, sensorimotor network; SUB, subcortical network; VAN, ventral attention network; VIS, visual network.

Next, we evaluated the prediction performance using subsets of networks and clinical metrics. First, we trained and tested models based on FC features from individual networks. Among the 9 networks analyzed, 3 demonstrated the most significant predictive ability: SMN (mean *r* = 0.19, *P* = .02), DMN (mean *r* = 0.19, *P* = .01), and CON (mean *r* = 0.26, *P* = .003 (Figure 4A and 4B). Second, we examined the effect of combining multiple networks on the prediction performance. As shown in Figure 4C, predictive performance gradually improved as more networks were incorporated into the model (eTable 1 in Supplement 1). A similar pattern was observed when progressively increasing the number of clinical metrics used as inputs, with enhanced prediction performance corresponding to the inclusion of additional clinical measures (Figure 4D; eTable 2 in Supplement 1). Notably, the performance of most subset models did not exceed that of the full model, suggesting that distributed FC patterns across the whole brain were generally more informative for predicting multidimensional treatment outcomes than models based on localized neural systems.

## Discussion

In this prognostic study, we developed and validated a transdiagnostic and transtherapeutic framework associating pretreatment whole-brain FC with multidimensional treatment outcomes in patients with IPs. Using the rCCA and a relatively large and heterogeneous sample of patients (N = 181) across 6 diagnostic categories and 3 treatment modalities, we found that whole-brain FC patterns significantly predicted symptom improvements across individuals, diagnoses, and treatments.

Although CBT, SSRI treatment, and ST engage different therapeutic mechanisms, the significant predictive power of our model suggested that successful symptom improvement may rely on shared neurobiological mechanisms. The cross-modality generalization may facilitate the development of predictive tools that inform treatment selection across different therapeutic approaches, reducing reliance on modality-specific assessments. For example, baseline FC patterns predictive of favorable CBT response may similarly identify individuals likely to benefit from SSRIs or ST, offering a path toward personalized interventions based on neurobiological markers. Similarly, our findings demonstrated cross-diagnosis generalization, supporting the notion of shared neurophysiological substrates across IPs. Models trained on one diagnostic group (eg, major depressive disorder) successfully predicted outcomes in other diagnostic groups. These results align with emerging frameworks, such as the Research Domain Criteria, which conceptualize mental disorders along dimensional constructs grounded in neurobiology rather than traditional categorical diagnoses.^35^

We identified distributed brain networks associated with symptom improvement following treatment for IPs. Specifically, connections positively contributing to the connectivity variate were predominantly observed within and between the default mode (DMN), frontoparietal (CON), and attention-related (DAN and VAN) networks. The DMN is a network largely implicated in internal mental processes, such as self-reflection and autobiographical memory, while the CON and the DAN and VAN are commonly activated during external oriented cognition. The interactions between these internally focused and externally directed circuits are crucial for cognitive and affective functions, with disruptions associated with psychopathology.^36^ Prior studies have shown that increased DMN coherence correlates with reduced maladaptive rumination and improved emotion regulation among patients undergoing CBT.^37^ Martens et al^38^ observed that treatment response for major depressive disorder was associated with increased FC between the right frontoparietal network and posterior DMN regions, suggesting that enhanced integration between these networks contributes to symptom improvement. A study by Posner et al^39^ showed that antidepressant treatment normalized this hyperconnectivity of the DMN in patients with dysthymia and that such normalization was associated with clinical improvement. Our results provide further evidence that higher baseline interactions between the DMN and task-related circuits may facilitate better treatment outcomes across diagnoses.

Connections negatively contributing to the connectivity variate were primarily observed within and between the DAN and VAN and lower-order networks, including the somatomotor network (SMN) and VIS. Accumulating evidence indicates that sensorimotor networks contribute to higher-order cognitive function and psychopathology.^40,41,42^ The FC of the SMN significantly contributes to multiple transdiagnostic dimensions of psychopathology.^42^ Dysconnectivity of the SMN and VIS is widely reported in psychiatric disorders, such as posttraumatic stress disorder, depression, and anxiety disorders.^43,44,45,46^ For example, patients with social anxiety disorder show reduced activation in occipital regions during attention- and emotion-related tasks,^47^ and visual cortex activation alterations are observed in anxiety disorders following both CBT and pharmacological treatments.^17^ Our findings extend prior work by suggesting that baseline FC patterns in then SMN and VIS are predictive of treatment outcomes, underscoring their relevance in neurobiological models of IPs.

Our analysis showed that predictive performance progressively improved as more clinical dimensions were integrated into the model, highlighting the value of multidimensional outcome assessment and their integration into computational tools and analytics. Psychiatric disorders often present with heterogeneous symptoms spanning emotional, cognitive, and somatic domains. Incorporating multiple clinical scales captures this complexity, improving both predictive accuracy and clinical relevance. For instance, both the Beck Depression Inventory and Hamilton Rating Scale for Depression assess depression but emphasize different domains: subjective emotional experience vs somatic symptoms, respectively. The Emotion Regulation Questionnaire evaluates emotion regulation strategies, while the Penn State Worry Questionnaire targets core features of worry in generalized anxiety disorder. Integrating such scales enables the identification of cross-dimensional symptom patterns, which is particularly valuable in transdiagnostic modeling. Our canonical loading analysis indicated that rumination (Ruminative Response Scale), worry (Penn State Worry Questionnaire), and social anxiety (Liebowitz Social Anxiety Scale) scales played a predominant role in driving the model’s prediction of symptom change. These findings align with prior evidence indicating that maladaptive repetitive thought processes, such as rumination and worry, are central transdiagnostic mechanisms across IPs. Both rumination and worry have been shown to contribute to the maintenance of depressive and anxious symptoms and are associated with altered connectivity within and between the DMN, salience network, and cognitive control networks.^48,49^ Likewise, social anxiety symptoms, as measured by the Liebowitz Social Anxiety Scale, have been associated with dysregulation in emotion-processing circuits, including the amygdala-prefrontal pathway and the visual-limbic pathway,^50,51^ which are also reflected in our connectivity findings.

Note that although ST is conventionally considered a control condition, our model achieved significant prediction performance in this group as well. This may be because our framework was designed to identify neural correlates of symptom change, rather than mechanisms specific to a particular treatment modality. Nevertheless, the identification of treatment-specific neural signatures remains an important goal. Future work incorporating posttreatment FC will enable the examination of how different interventions uniquely alter brain connectivity patterns.

### Limitations

Limitations of this study should be considered. First, there is a limitation regarding the specificity of these predictors. Our results do not show specific predictions within a given treatment modality (CBT vs pharmacotherapy, for example). The number of participants assigned to each treatment group was relatively small, which precluded a reliable assessment of treatment-specific prediction. Second, the model demonstrated robust performance across different cross-validation procedures in a relatively large dataset, but the generalizability of our findings beyond this sample remains to be tested. Although the model demonstrated statistically significant prediction of multidimensional symptom improvement, the explained variance at the individual level was modest. This finding reflects a common challenge in brain-based prediction studies and suggests that the current model, while informative, does not account for a substantial portion of individual variability in treatment response. The modest effect size likely reflects the complex, multifactorial nature of clinical outcomes and the inherent noise in both neural and behavioral measurements. Nevertheless, we believe that even moderate predictive power can reveal meaningful brain-behavior associations and provide a foundation for identifying reliable biomarkers. Future work should validate these connectivity-symptom associations in independent, larger, and more balanced cohorts and incorporate before and after treatment contrasts to isolate modality-specific neural change. Integrating multimodal data, more fine-grained behavioral phenotyping, and larger samples may improve prediction performance.

## Conclusions

In this prognostic study assessing predictive modeling of 181 patients with IPs, pretreatment whole-brain FC predicted multidimensional symptom change and generalized across diagnostic categories and treatment modalities. The most influential connections clustered within the DMN and the DAN and VAN, and predictive performance decreased when either neural systems or clinical dimensions were reduced. These findings suggest that transdiagnostic connectivity signatures may inform personalized treatment planning for patients with IP.

## Notes

Supplement 1.
eMethods.
**eTable 1.** Prediction Performance on Different Network Combination **eTable 2.** Prediction for Different Dimensional Clinical Metrics **eFigure 1.** Transdiagnostic-Therapeutic Prediction Modeling for Treatment Outcome **eFigure 2.** Predictive Performance Based on 1-PC Step Grid Search **eFigure 3.** Predictive Performance for Each Canonical Variate Pair Based on Permutation Test **eFigure 4.** The Correlation Between Connectivity Variates and Clinical Metric in Different Transdiagnostic Connectome Based on Prediction Strategies **eFigure 5.** Distribution of Loading in Brain Side and Symptom Side Across 1000 Bootstrap Samples **eFigure 6.** Distribution of Canonical Variates for Top-Tier Responders and Lower-Tier Responders

Supplement 2.
Data Sharing Statement
