# Supplementary material for: Functional Connectivity Predicting Transdiagnostic Treatment Outcomes in Internalizing Psychopathologies
Source: JAMA Netw Open. 2025 Sep 3;8(9):e2530008. doi: 10.1001/jamanetworkopen.2025.30008 (PMC12409597; doi:10.1001/jamanetworkopen.2025.30008)
Supplement: Supplement 1. — eMethods. eTable 1. Prediction Performance on Different Network Combination eTable 2. Prediction for Different Dimensional Clinical Metrics eFigure 1. Transdiagnostic-Therapeutic Prediction Modeling for Treatment Outcome eFigure 2. Predictive Performance Based on 1-PC Step Grid Search eFigure 3. Predictive Performance for Each Canonical Variate Pair Based on Permutation Test eFigure 4. The Correlation Between Connectivity Variates and Clinical Metric in Different Transdiagnostic Connectome Based on Prediction Strategies eFigure 5. Distribution of Loading in Brain Side and Symptom Side Across 1000 Bootstrap Samples eFigure 6. Distribution of Canonical Variates for Top-Tier Responders and Lower-Tier Responders [file jamanetwopen-e2530008-s001.pdf]

Supplementary Online Content

Zhang K, Klumpp H, Jimmy, J, Phan KL, MD, Milad MR, Wen Z. Functional Connectivity Predicting Transdiagnostic Treatment Outcomes in Internalizing Psychopathologies. *JAMA Netw Open*. 2025;8(9):e2530008. doi:10.1001/jamanetworkopen.2025.30008

eMethods.

eTable 1. Prediction Performance on Different Network Combination

eTable 2. Prediction for Different Dimensional Clinical Metrics

eFigure 1. Transdiagnostic-Therapeutic Prediction Modeling for Treatment Outcome

eFigure 2. Predictive Performance Based on 1-PC Step Grid Search

eFigure 3. Predictive Performance for Each Canonical Variate Pair Based on Permutation Test

eFigure 4. The Correlation Between Connectivity Variates and Clinical Metric in Different Transdiagnostic Connectome Based on Prediction Strategies

eFigure 5. Distribution of Loading in Brain Side and Symptom Side Across 1000 Bootstrap Samples

eFigure 6. Distribution of Canonical Variates for Top-Tier Responders and Lower-Tier Responders

eReferences.

This supplementary material has been provided by the authors to give readers additional information about their work.

© 2025 Zhang K et al. *JAMA Network Open*.

eMethods.

The Inclusion and Exclusion of Participants Recruitment

For clinical trial 1, the exclusion criteria for patients were as follows: inability to provide informed consent or to read and write in English; a lifetime history of manic or hypomanic episodes, psychotic symptoms, traumatic brain injury, intellectual disability, pervasive developmental disorders, or dementia; active suicidal ideation; alcohol or substance dependence within the past six months; concurrent pharmacotherapy or psychotherapy at the time of enrollment and throughout the study; pregnancy; and MRI contraindications. Additionally, participants were required to have no contraindications to psychiatric treatment, no history of failure to respond to more than two SSRI trials, and no primary psychopathology unsuitable for the treatment algorithm (e.g., obsessive-compulsive disorder due to higher SSRI dosage requirements, adjustment disorders, and specific phobias for which SSRIs are not indicated. To be eligible for inclusion in the patient group, participants were required to meet full- or sub-threshold (i.e., all but one criteria) diagnostic criteria for a DSM-5 depression, anxiety, or posttraumatic stress disorder, a total score of 23 or higher on the Depression, Anxiety, and Stress Scale (DASS-21), and a Global Assessment of Functioning score of 60 or lower. The total score of $\geqslant$ 23 threshold for the DASS-21 was chosen as this indicates a level of psychopathology that warrants treatment.

For clinical trial 2, the exclusion criteria for patients were major active medical or neurological problems; past or current major psychiatric illness (e.g., bipolar disorder, schizophrenia); active suicidal ideation or non-suicidal self-injury in the last 6 months; use of psychotropic medication in the 6 weeks prior to study entry or during the study; cognitive dysfunction (e.g., traumatic brain injury, dementia, intellectual disability); pervasive developmental disorder (e.g., autism,

© 2025 Zhang K et al. *JAMA Network Open*.

learning disability); substance abuse or dependence in the last 6 months; contraindications for functional magnetic resonance imaging (fMRI) (e.g., ferrous metal, pregnancy, claustrophobia); and a positive toxicology test before the MRI scan. To be eligible for inclusion, all participants were required to be between 18- and 65- years old and have a diagnosis of SAD (n = 39) or MDD (n = 33) based on DSM-5 diagnostic criteria. Patients were also required to either surpass a symptom severity threshold (i.e., a Liebowitz Social Anxiety Scale (LSAS) score ⩾60 for SAD patients; a Hamilton Depression Rating Scale (HAMD) score ⩾17 or a Beck Depression Inventory-II score ⩾16 for MDD patients) or report clinically significant levels of impairment (based on diagnostic interview).

Participants

All the data used for analyzing are from participants (n = 181) aged 18 to 65 years with no major medical or neurologic illness as confirmed by a Board-Certified physician. Comorbid diagnoses were social anxiety disorder (37.5%, n=68), major depressive disorder (37.5%, n=68), generalized anxiety disorder (19.3%, n=35), post-traumatic stress disorder (0.027% n=5), panic disorder (0.022% n=4), persistent depressive disorder (0.005% n=1).

Treatment Procedures

All treatment procedures and data collection were conducted at the UIC. Data collection ceased upon reaching the predetermined sample size. Participants were randomized to treatment arms through simple randomization using a random number generator, and assignments were made by research coordinators.

© 2025 Zhang K et al. *JAMA Network Open*.

For clinical trial 1, patients were randomized to assigned to SSRI or CBT arm. For the SSRI arm, participants attended 20-30 minute medication management sessions with their study psychiatrist at weeks 0, 2, 4, 8, and 12[1]. The SSRI dosing regimen followed standard psychiatric practice, allowing the study physician to choose the specific SSRI and design a flexible dosing schedule based on the participant's previous medication history. Medications included sertraline (50-200 mg), citalopram (10-40 mg), escitalopram (5-20 mg), paroxetine (10-60 mg), and fluoxetine (10-80 mg), with the aim of achieving a target dose by week 8, to be maintained for the final four weeks. For the CBT arm, treatment consisted of 60-minute sessions held once weekly for 12 weeks, delivered by a Ph.D. level clinical psychologist using evidence-based treatment manuals tailored to the participant's primary diagnosis and predominant symptoms. Sessions were supervised by a senior CBT therapist and researcher (MGC) to ensure treatment fidelity and adherence. The therapy followed manualized protocols, beginning with psychoeducation and orientation to CBT, progressing to cognitive restructuring and behavioral interventions (e.g., exposure exercises, behavioral activation), and concluding with relapse prevention strategies. Psychiatric comorbidity and the frequent overlap between depression and anxiety were addressed both during psychoeducation and throughout the treatment.

For clinical trial 2, patients were randomized to receive 12 weekly 60-minute sessions of either CBT or ST using a covariate adaptive randomization approach[2]. Those assigned to the CBT group received structured, manualized therapy tailored for either SAD or MDD. The sessions focused on core therapeutic components, including psychoeducation, cognitive restructuring, behavioral activation (for MDD), in vivo exposure exercises (for SAD), and relapse prevention strategies. In contrast, ST followed a client-centered therapy approach[3], emphasizing reflective listening and the facilitation of emotional expression as appropriate.

© 2025 Zhang K et al. *JAMA Network Open*.

Neuroimaging Data Acquisition and Processing

Neuroimaging data were collected using a 3T GE Discovery System MR scanner (General Electric, Waukesha, WI). High-resolution T1-weighted (T1w) volumetric anatomical scans were obtained for spatial registration. Resting-state and task-based fMRI data were acquired using gradient echo-planar imaging (EPI) with the following parameters: 44 axial slices, repetition time (TR) = 2 s, flip angle = 90°, echo time (TE) = 25 ms, field of view (FOV) = 220 x 220 mm, acquisition matrix = 64 x 64, slice thickness = 3 mm with no gap, and voxel size = 3.4 x 3.4 x 3 mm.

Preprocessing was conducted using fMRIPrep 20.0.2, incorporating standard spatial normalization, motion correction, nuisance regression, and temporal filtering procedures. The T1w images underwent intensity non-uniformity correction using N4BiasFieldCorrection (ANTs 2.3.3) and served as the T1w reference for all subsequent steps. This T1w reference was then skull-stripped, segmented into cerebrospinal fluid, white matter, and gray matter, and spatially normalized to Montreal Neurological Institute (MNI) space (MNI152NLin2009cAsym) using nonlinear registration with antsRegistration (ANTs 2.3.3). Functional images were corrected for head motion with mcflirt (FSL) and adjusted for slice-timing using 3dTshift (AFNI). Next, these preprocessed functional images were co-registered to the T1w reference with boundary-based registration (nine degrees of freedom) using flirt (FSL) and subsequently normalized to the MNI152NLin2009cAsym space by applying the spatial normalization parameters derived from the T1w reference. After normalization, the functional images were resampled to a voxel size of 2 × 2 × 2 mm using Lanczos interpolation (ANTs 2.3.3) and subsequently smoothed with a Gaussian kernel having a 6-mm full-width at half-maximum. The following nuisance covariates, including six head motion parameters, averaged white matter signal, averaged cerebral spinal

© 2025 Zhang K et al. *JAMA Network Open*.

fluid (CSF) signal, their temporal derivatives, quadratic terms, and squares of derivatives were regressed out from the brain signals. The brain time series were band-pass (between 0.01 and 0.08 Hz) filtered to reduce low-frequency drift and high-frequency noise.

fMRI Paradigms

In clinical 1, the data analyzed included four task-state fMRIs and one resting-state fMRI. Among them, the task state includes fear extinction (EXT), recall of fear (REC), the emotion regulation task (ERT) and emotional faces interference task (EFIT). Runs included 150 and 240 T2*-weighted functional images, respectively. In clinical 2, the data analyzed included two task-state fMRIs (ERT, EFIT) and one resting-state fMRI.

For the first two task, participants underwent a validated 3-day fear conditioning and extinction paradigm[4–6]. During the conditioning phase, participants were exposed to three cues (conditioned stimuli, CS), where two cues (CS+) were partially reinforced with mild electric shocks, while the third cue (CS-) was never paired with shocks. Notably, no fMRI data was collected during this phase. In the extinction learning phase, participants were repeatedly presented with one of the CS+ and the CS- cues without receiving any shocks. Finally, in the recall phase, participants were exposed to all three cues: the extinguished CS+ (CS+E), the unextinguished CS+ (CS+U), and the CS-. fMRI data collection was performed during the latter two phases of the task.

For ERT[7,8], participants viewed negative and neutral images from the International Affective Picture System (IAPS) under three conditions : (1) “Look Neutral,” where they viewed neutral images without altering their emotional response; (2) “Look Negative,” where they viewed negative images naturally without attempting to modify their emotional response; and (3)

© 2025 Zhang K et al. *JAMA Network Open*.

"Reappraise Negative," where they reduced the emotional impact of negative images by interpreting the depicted scenario in a less negative manner.

For EFIT[9], Participants were briefly shown a string of six letters superimposed on a task-irrelevant face distractor and instructed to identify target letters (N or X) as quickly and accurately as possible via button press. The distractor faces displayed fearful, angry, or neutral expressions, selected from a standardized stimulus set featuring eight different individuals. During low perceptual load trials, all six letters in the string were target letters, while high perceptual load trials included one target letter alongside five non-target consonants (e.g., HKMWZ) presented in random order. The task was completed over two image acquisition runs, each consisting of 12 blocks with 5 trials per block. A mixed block/event-related design was employed, with perceptual load (low vs. high) alternating between blocks, while facial expressions varied randomly on a trial-by-trial basis within each block. Each image was displayed for 200 ms, followed by a fixation cross for 1800 ms. The intertrial interval ranged from 2 to 6 seconds within blocks, and blocks were separated by intervals of 4 to 8 seconds.

In our analysis, we ignored the task information and treated all fMRI as task-irrelevant state.

Estimation of Functional Connectivity Matrix

To construct the functional connectivity (FC) matrix, we used a 442-region whole-brain atlas comprising 400 cortical regions[10] , 32 subcortical regions[11] , and 10 cerebellar regions[12]. Regional time series were extracted by averaging the time series across voxels within each region. To reduce the influence of head motion, time points with framewise displacement (FD) larger than 0.3 mm were censored. For each participant, FC matrices (442x442) were first computed separately for each fMRI run (resting-state and task-based) using pairwise Pearson

© 2025 Zhang K et al. *JAMA Network Open*.

correlation. These run-level matrices were then averaged to generate a single connectivity matrix per participant, which was used in following analyses. The upper triangular elements of the mean matrix (excluding the diagonal) which including 442×442/2=97,461 connections (edges) were used for subsequent predictive analyses.

Regularized Canonical Correlation Analyses with Cross Validation

To investigate associations between functional connectivity (FC) and clinical measures, we conducted regularized canonical correlation analysis (rCCA), which is a multivariate statistical method designed to identify linear combinations of two sets of variables that maximally correlate with each other. This method is particularly suitable for high-dimensional data, where the number of features exceeds the number of observations.

1. Functional Connectivity Feature Extraction

We computed the functional connectivity (FC) values between 442 predefined brain regions, generating symmetric region-by-region correlation matrices (442 × 442) for each participant. These FC matrices were calculated based on the Pearson correlation of preprocessed time series extracted from each region of interest (ROI).

To convert the symmetric FC matrices into feature vectors suitable for machine learning modeling, we used the *'sym_matrix_to_vec'* function provided by the Nilearn library (version 0.11.1). This function extracts the upper triangular elements (excluding the diagonal) of each symmetric matrix and flattens them into one-dimensional vectors. This procedure ensures that the redundant, mirrored lower triangular part of the matrix is not included, reducing computational complexity while preserving the full information content of the functional connectivity relationships.

© 2025 Zhang K et al. *JAMA Network Open.*

As a result, each participant's FC matrix was transformed into a feature vector of 97,461 unique edgewise connections (computed as [442 × (442 - 1)] / 2), which served as the input for subsequent dimensionality reduction and predictive modeling analyses.

2. Dimensionality Reduction using PCA

Given the high dimensionality of the FC feature space, we applied Principal Component Analysis (PCA) for dimensionality reduction prior to rCCA. PCA was implemented using the PCA function from the Scikit-learn library. For the selection of principal components (PCs), large-scale connectome–behavior studies adopt the top PCs that explaining major variance before entering multivariate analysis[13–15]. Therefore, we define the selection range of PCs from 5~ 30 and then applied grid search to select the optimal parameters (Grid search strategy see below).

3. Data Standardization of Clinical Measures

Clinical measures, including BDI, HAMA, HAMD, PSWQ, LSAS, RUM, ERQ suppression, and ERQ reappraisal, were standardized using the *StandardScaler* from Scikit-learn. The scaler was fit on the training data in each outer fold and applied to both the training and test sets, ensuring consistent scaling and preventing data leakage. Standardization was performed by centering the data (zero mean) and scaling to unit variance based on the training set statistics.

4. Fitting the rCCA Model

We then fit the rCCA model to assess the associations between the dimensionality-reduced functional connectivity features and clinical metrics. Conceptually, rCCA identifies linear combinations of the connectivity and clinical variables—referred to as the connectivity variate and clinical variate, respectively—such that the correlation between them is maximized.

© 2025 Zhang K et al. *JAMA Network Open*.

The rCCA implementation was performed using the rCCA function from the CCA-Zoo Python library (version 2.6.0), which provides functional connectivity features and the clinical metrics —such that the connectivity variate and clinical variate is maximized.

Compared to standard CCA, rCCA incorporates an L2 regularization penalty on the covariance matrices of the two datasets, enhancing model stability and preventing overfitting, particularly in high-dimensional settings where the number of features exceeds the number of samples. In our study, the regularization parameter c was optimized through an inner cross-validation loop, with values ranging from 0 to 1 in increments of 0.05.

5. Outer Cross-Validation Framework

To evaluate the generalizability and robustness of the rCCA model, we implemented a nested cross-validation procedure comprising multiple validation strategies (eFigure 1):

a) Cross-Individual Validation

We conducted 10-fold cross-validation, repeated five times, across all participants. In each iteration, nine folds were used for training and one fold for testing. The rCCA model was trained on the training data, and the canonical weights learned were applied to the test data to predict connectivity and clinical variates.

b) Cross-Diagnosis Validation

To test the generalizability across different diagnostic groups, we used a leave-one-diagnosis-out strategy. We divided the full sample into six non-overlapping diagnostic groups, based on participants' primary diagnoses. In each cross-validation iteration, five diagnostic groups were

© 2025 Zhang K et al. *JAMA Network Open*.

used as training data, and the hold-out diagnostic group was used as testing data. The model was trained on the training data and applied on the testing data.

c) Cross-Treatment Modalities Validation

To explore the generalization of predictive models across different therapeutic interventions, we conducted a cross-treatment validation. In each iteration, data from two out of three treatment modalities (CBT, SSRI, ST) were used for training, and the third was used as the test set. This process was repeated for each treatment modality left out, and the results were reported separately for each condition.

6. Inner Cross-Validation for Parameters Selection

In the outer loop, the full sample was divided into outer training data and testing data according to different validation strategies, i.e., standard k-fold cross-validation, cross-diagnosis validation, or cross-treatment modalities validation. Within the inner loop, an inner 5-fold cross-validation procedure was performed on the outer training data. Specifically, the outer training data was randomly divided into 5 inner folds. For each candidate hyperparameter (PCA components, regularization parameter), the model was trained on four inner folds and tested on the hold-out inner fold. This was repeated across all five inner folds, and predictive performance was estimated by calculating the Pearson correlation between predicted connectivity and clinical canonical variate scores across participants in the inner folds.

The number of PCs and regularization parameter that resulted in the best prediction performance in the inner loop was selected as the optimal number. And then, the model was retrained on the entire outer training data using this selected hyperparameter and applied to the outer testing data.

© 2025 Zhang K et al. *JAMA Network Open*.

This nested cross-validation procedure ensured that hyperparameter tuning was restricted to the training data only, thereby providing an unbiased estimate of generalization performance.

7. Permutation Testing for Significance Assessment

We used a permutation test to evaluate the significance of the cross-validation results. The clinical measures were randomly shuffled across participants, and the 5-fold cross-validation procedure was repeated 1000 times to obtain a null distribution of the correlation values between the predicted connectivity and clinical variants. The significance was determined by comparing the observed correlation to the null distribution.

8. Canonical Loadings Analysis

We extracted the canonical loadings using the repeated 10-fold cross-validation. Specifically, in each fold of the 10-fold cross-validation, we trained the rCCA model on the training data and applied it to the hold-out testing data to predict the canonical variate scores (one for the connectivity variate, the other for the clinical variate) for each participant in the testing data. This process was repeated across all 10 folds, such that each participant had predicted canonical variate scores obtained from a fold where they were in the testing data. To compute the connectivity loadings, we calculated the Pearson correlation between the full vector of predicted connectivity variate scores and each connectivity feature across participants. Connectivity loadings that survived FDR correction ($p < 0.05$) were visualized in Figure 3. Similarly, symptom loadings were computed as the Pearson correlation between the predicted symptom variate scores and each clinical symptom measure. This cross-validated approach ensures that the loadings reflect relationships between model-derived canonical variates and input features in

© 2025 Zhang K et al. *JAMA Network Open*.

held-out data. Besides, a non-parametric bootstrap (1000 iterations) procedure was used to assess the repeatability and stability of significant connections and clinical-scale loadings.

Prediction Using a Subset of Networks and Clinical Metrics

In the main analysis, we used whole-brain FC patterns to predict the treatment outcomes. This approach was informed by emerging evidence suggesting that distributed neural systems are integrated for diverse cognitive and emotional functions[16–18], thus may better capturing individual variability in treatment response compared to localized neural patterns. To examine this, we conducted additional post-hoc analyses by restricting the predictive model to connections within specific networks or between subsets of networks. We systematically constructed predictive models for all possible network combinations and evaluated their respective predictive performances.

To assess the impact of clinical metric dimensionality on predictive accuracy, we examined the effect of using a single vs. multiple clinical metrics. While individual clinical scales focus on specific symptom domains, they may not fully capture multidimensional symptom change. In contrast, integrating multiple scales may provide a more comprehensive representation of clinical symptoms and treatment response, potentially enhancing predictive performance and model generalizability. We conducted a comparative analysis by progressively modifying the input from a single clinical metric to multi-metric combinations, ultimately incorporating all eight clinical scales. The predictive performance was then assessed across different levels of clinical dimensionality.

© 2025 Zhang K et al. *JAMA Network Open*.

eResults.

The Influence of Principal Component Step Size on Prediction Performance

To explore how does the PCs step size will influence the predictive performance and verify that the selection of 5-PC increment did not bias model selection, we repeated the entire inner-loop search using a 1-PC step within the same nested 10-fold cross-validation.

The predictive performance (eFigure 2) revealed that predictive performance based on 1-PC step grid search strategy keeps the similar powerful performance compared to 5-PC step. Therefore, we believe that 5 PCs is the optimal step size parameter choice that ensures both performance and computational efficiency.

Selection of the Canonical Covariate Pairs

Multiple canonical variate pairs could be estimated using rCCA model (up to 8 in our case). To identify the canonical variate pairs that represent the generalizable brain-symptom associations, we used a permutation test procedure (1000 times of shuffling). Specifically, we randomly shuffled the clinical measures across participants (row-wise) and conducted a 10-fold cross-validation as described in the main text. Predictive performance was estimated for each of the eight canonical variate pairs. This procedure was repeated 1000 times to generate a null distribution of predictive performance for each canonical pair. The predictive performances obtained using the original dataset were separately tested against the null distributions. As shown in eFigure 3, the first canonical variate pair was the only one with statistically significant performance ($r = 0.37$, $p < 0.01$), while all other pairs failed to exceed chance level (all $p > 0 .10$).

Prediction Performance with Percentage Change of Clinical Metric

© 2025 Zhang K et al. *JAMA Network Open*.

To further validate the robustness of our predictive model, we computed the outcome variable as the percentage change in clinical scores[9] from baseline to post-treatment, defined as:

$$Percentage\ chagne = \frac{Pre\ treatment\ Score - Post\ treatment\ Score}{Pre\ treatment\ Score}$$

Using this normalized metric, we repeated the predictive analyses across the same validation schemes. Consistent with our primary findings, the rCCA model significantly predicted percentage symptom change from pre-treatment functional connectivity features. Under 10-fold standard cross-validation, the mean correlation between predicted and observed percentage change scores was r =0.36, p = 0.009 (eFigure 4A). Similarly, in cross-diagnosis validation, where models were trained on participants from five diagnostic subtypes and tested on the left-out group, predictive performance remained significant (r =0.22, p = 0.03; eFigure 4B). These results further demonstrate the robustness of our predictive framework, indicating that functional connectivity patterns reliably capture treatment-related symptom change regardless of how such change is quantified.

Prediction Performance Using a Subset of Networks and Clinical Metrics

To examine how the dimensionality of both neural networks (i.e., number of networks) and clinical metric (i.e., dimensionality of scales) influences predictive performance, we performed additional combinatorial analyses.

First, we systematically evaluated prediction performance when using different combinations of brain networks to predict the full set of clinical scales. As shown in eTable 1, predictive accuracy increased progressively with the number of included networks. While single-network models

© 2025 Zhang K et al. *JAMA Network Open*.

yielded limited performance, combining multiple networks—particularly when approaching whole-brain coverage—led to substantial improvements, with the highest performance achieved using all nine networks.

Second, we assessed the impact of varying clinical metric dimensionality, using full-brain connectivity features to predict different combinations of symptom scales. As reported in eTable 2, models trained on a greater number of clinical measures achieved increasingly better prediction accuracy, demonstrating a clear cumulative effect. Importantly, models using only a single metric often failed to reach significance, while models using multiple dimensions—particularly those combining affective, cognitive, and regulatory symptom domains—produced stronger and more stable predictions.

In addition, eTable 2 revealed that among all possible combinations of clinical metrics, the PSWQ, LSAS, and RUM appeared most frequently in the top-performing combinations that yielded the highest prediction accuracy. This observation is consistent with our canonical loading analysis reported in the main text, which identified RUM, PSWQ, and LSAS as the clinical measures contributing most strongly to the clinical canonical variate. These converging findings suggest that repetitive negative thinking, social anxiety, and pathological worry represent core symptom domains that are most tightly coupled with distributed brain connectivity patterns predictive of treatment-related change.

Stability Evaluation Using Bootstrap

To evaluate the stability of both canonical variates, we performed a non-parametric bootstrap procedure. Within each of the 1000 iterations, participants were resampled with replacement. For

© 2025 Zhang K et al. *JAMA Network Open.*

each resample, we refit the rCCA model and calculated the correlations between the canonical variates and individual features on the brain-side and symptom-side. The results were shown as follows:

As shown in eFigure 5, the bootstrapped contributions closely matched the original solution. On the brain side, resampled connectivity patterns also preserved the original pattern, including positively weighted edges linking the default mode to dorsal/ventral attention and frontoparietal control networks, and negatively weighted edges linking the sensorimotor and visual networks. On the symptom side, the relative contribution of each clinical measure was preserved, with rumination (median $r = 0.66$), worry (PSWQ, median $r = 0.63$), and social anxiety (LSAS, median $r = 0.61$) remaining the strongest positive contributors.

Distribution of Treatment Responders versus Treatment Non-Responders in Prediction

To explore the clinical interpretation of the canonical clinical variate, we performed an exploratory responder analysis. The three symptom measures showed the highest correlations with the clinical variate (LSAS, PSWQ, and RUM) were used to divide participants into top-tier responders and lower-tier responders. Specifically, for each participant, we first z-scored the change scores (pre-post) on LSAS, PSWQ, and RUM across subjects, and averaged the three z-scores to generate a composite treatment-response index. Participants were then stratified into top-tier responders and lower-tier responders using a median split of this index. We compared the predicted canonical symptom scores between the two responder groups using a two-sample t-test. Results showed that top-tier responders had significantly higher predicted canonical clinical variates than lower-tier responders ($p < 0.005$; eFigure 6). Similar results were obtained when the top-tier responders and lower-tier responders were defined based on each individual symptom

change score (LSAS, PSWQ, or RUM; all $p < 0.005$). These findings support the clinical relevance of the identified canonical variates.

© 2025 Zhang K et al. *JAMA Network Open*.

eTable 1. Prediction Performance on Different Network Combination

**Number of Networks**	**Average *r* value**	**Network Combination for Best Predictive Performance**
1	0.09	CON
2	0.15	CON, DMN
3	0.20	VIS, CON, DMN
4	0.24	VIS, CON, DMN, SUB
5	0.27	VIS, CON, DMN, SUB, SMN
6	0.30	VIS, CON, DMN, SUB, SMN, DAN
7	0.32	VIS, CON, DMN, SUB, SMN, DAN, VAN
8	0.35	VIS, CON, DMN, SUB, SMN, DAN, VAN, CEM

eTable 2. Prediction for Different Dimensional Clinical Metrics

**Dimensionality of Clinical Metrics**	**Average *r* value**	**Metric Combination for Best *r* value**
1	0.09	LSAS
2	0.16	LSAS， RUM
3	0.20	PSWQ, LSAS, RUM
4	0.25	HAMD, PSWQ, LSAS, RUM
5	0.28	HAMD, PSWQ, LSAS, RUM, ERQ-S
6	0.31	BDI, HAMD, PSWQ, LSAS, RUM, ERQ-S
7	0.34	BDI, HAMA, HAMD, PSWQ, LSAS, RUM, ERQ-S

© 2025 Zhang K et al. *JAMA Network Open*.

eFigure 1. Transdiagnostic-Therapeutic Prediction Modeling for Treatment Outcome

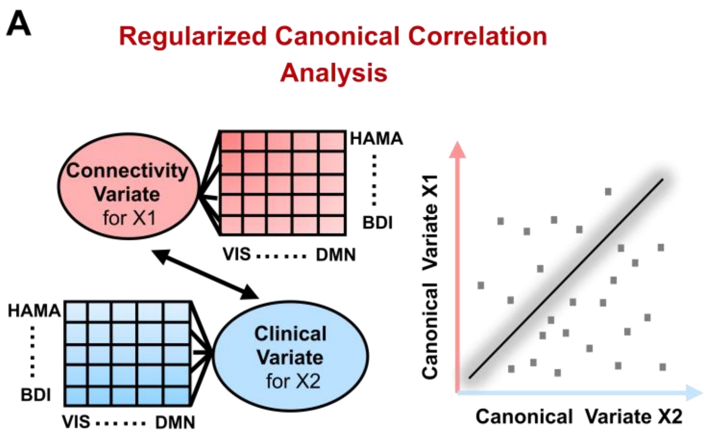

© 2025 Zhang K et al. *JAMA Network Open*.

eFigure 2. Predictive Performance Based on 1-PC Step Grid Search

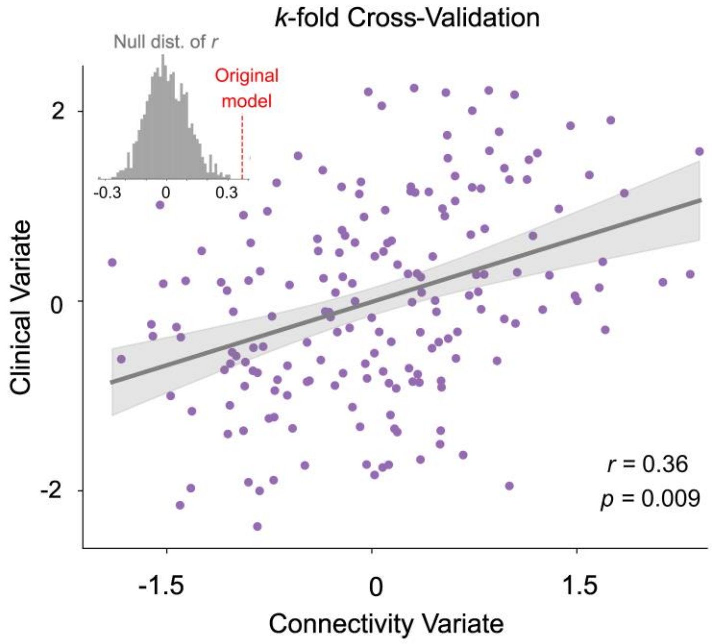

© 2025 Zhang K et al. *JAMA Network Open*.

eFigure 3. Predictive Performance for Each Canonical Variate Pair Based on Permutation Test. (The red vertical lines represent predictive performance based on the original dataset. The histograms represent null distributions obtained using permutation tests.)

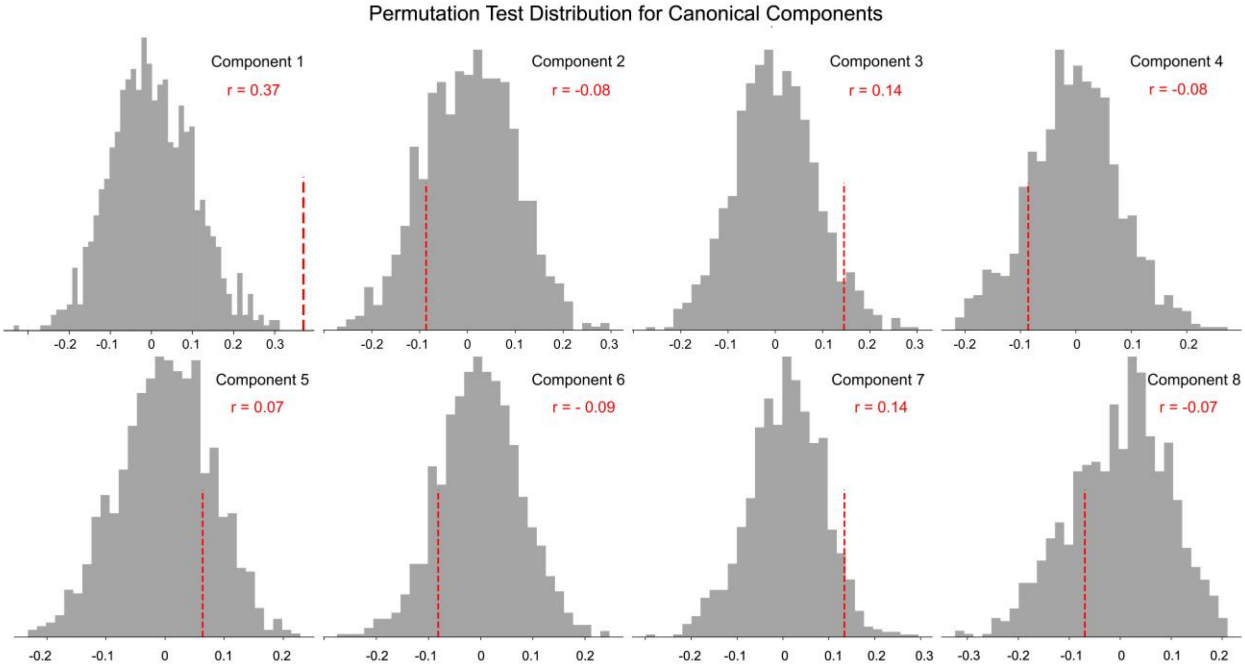

© 2025 Zhang K et al. *JAMA Network Open*.

eFigure 4. The Correlation Between Connectivity Variates and Clinical Metric in Different Transdiagnostic Connectome Based on Prediction Strategies. (A). Across participant (B). Across diagnosis.

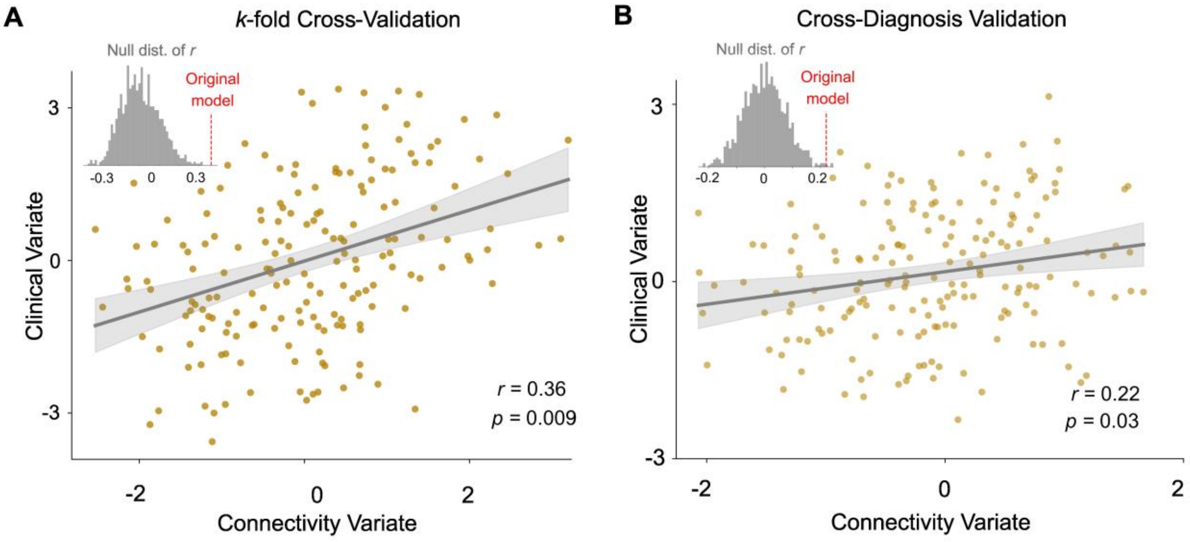

© 2025 Zhang K et al. *JAMA Network Open*.

eFigure 5. Distribution of Loading in Brain Side and Symptom Side Across 1000 Bootstrap Samples. (A). The connectivity mechanism between positive connectivity and symptom change. (B). The connectivity mechanism between negative connectivity and symptom change. (C). The canonical loadings of the clinical metrics represent the contribution of each metric for prediction.

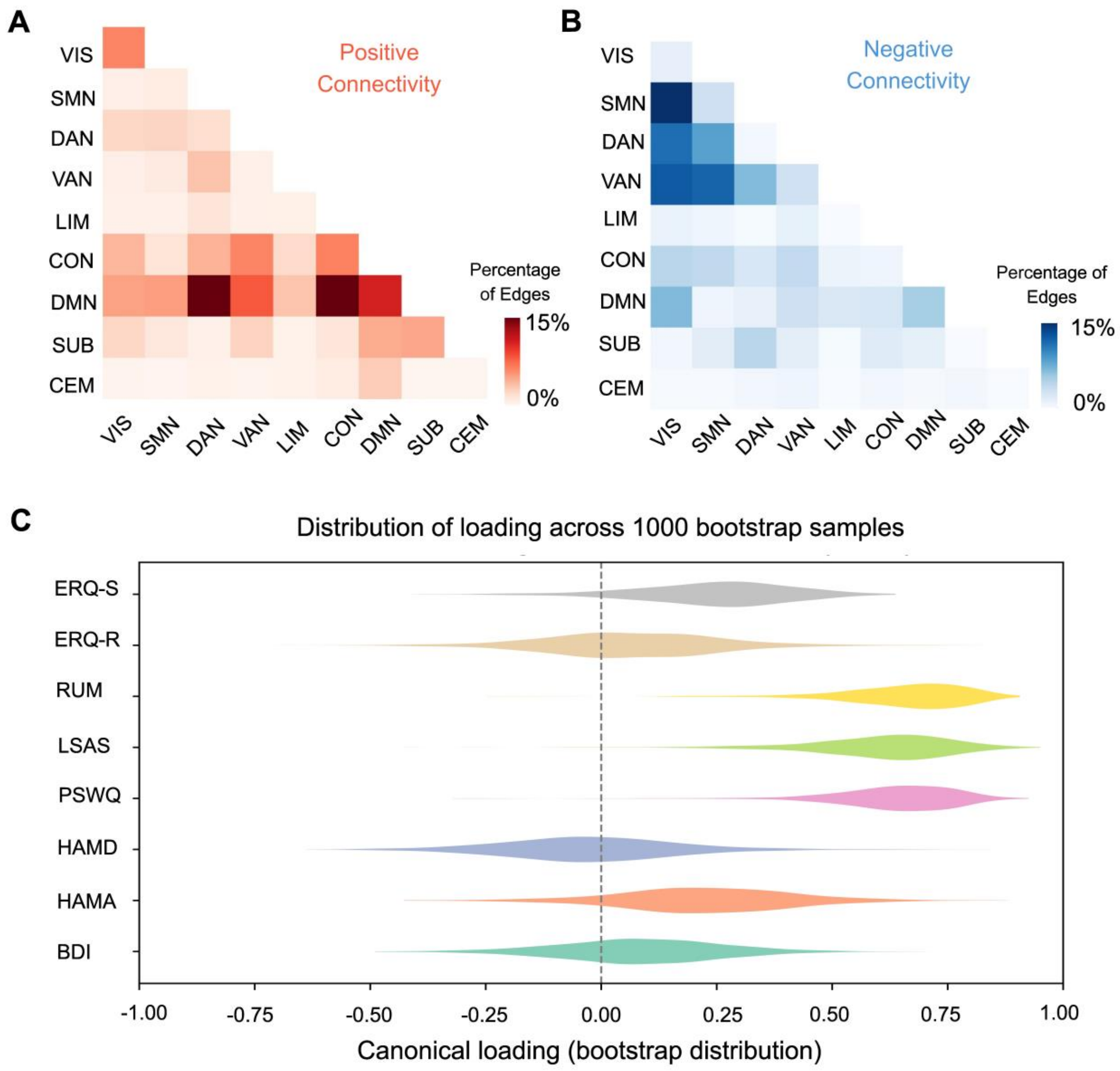

© 2025 Zhang K et al. *JAMA Network Open*.

eFigure 6. Distribution of Canonical Variates for Top-Tier Responders and Lower-Tier Responders. Liebowitz Social Anxiety Scale (LSAS), Penn State Worry Questionnaire (PSWQ), and Ruminative Response Scale (RUM) were used to define top-tier responders (blue dots) and lower-tier responders (yellow dots).

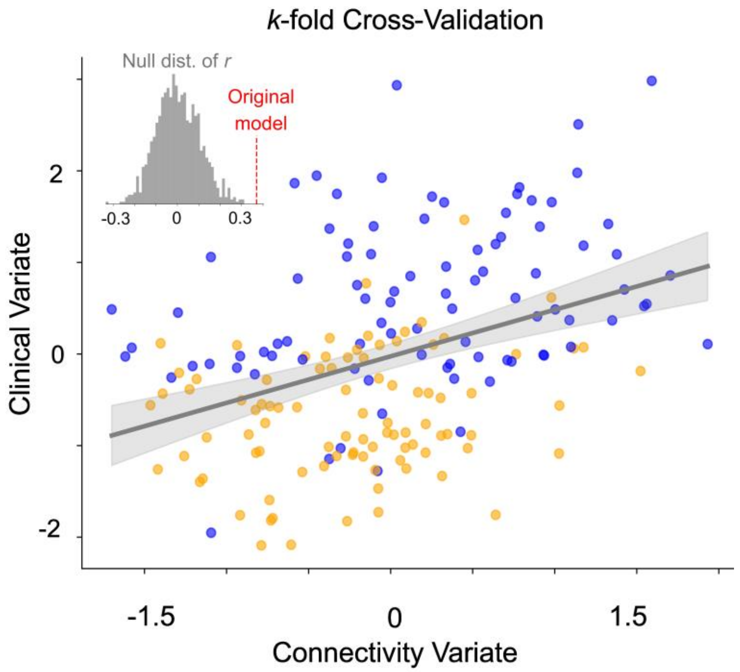

© 2025 Zhang K et al. *JAMA Network Open*.

eReferences

1. Gorka SM, Burkhouse KL, Klumpp H, et al. Error-related Brain Activity as a Treatment Moderator and Index of Symptom Change during Cognitive-Behavioral Therapy or Selective Serotonin Reuptake Inhibitors. *Neuropsychopharmacol*. 2018;43(6):1355-1363. doi:10.1038/npp.2017.289

2. Feurer C, Jimmy J, Uribe M, et al. Brain activity during reappraisal and associations with psychotherapy response in social anxiety and major depression: a randomized trial. *Psychol Med*. Published online May 22, 2024:1-11. doi:10.1017/S0033291724001120

3. Markowitz JC, Manber R, Rosen P. Therapists' Responses to Training in Brief Supportive Psychotherapy. *APT*. 2008;62(1):67-81. doi:10.1176/appi.psychotherapy.2008.62.1.67

4. Milad MR, Pitman RK, Ellis CB, et al. Neurobiological Basis of Failure to Recall Extinction Memory in Posttraumatic Stress Disorder. *Biological Psychiatry*. 2009;66(12):1075-1082. doi:10.1016/j.biopsych.2009.06.026

5. Wen Z, Seo J, Pace-Schott EF, Milad MR. Abnormal dynamic functional connectivity during fear extinction learning in PTSD and anxiety disorders. *Mol Psychiatry*. 2022;27(4):2216-2224. doi:10.1038/s41380-022-01462-5

6. Milad MR, Wright CI, Orr SP, Pitman RK, Quirk GJ, Rauch SL. Recall of fear extinction in humans activates the ventromedial prefrontal cortex and hippocampus in concert. *Biol Psychiatry*. 2007;62(5):446-454. doi:10.1016/j.biopsych.2006.10.011

7. Klumpp H, Jimmy J, Burkhouse KL, et al. Brain response to emotional faces in anxiety and depression: neural predictors of cognitive behavioral therapy outcome and predictor-based subgroups following therapy. *Psychological Medicine*. 2022;52(11):2095-2105. doi:10.1017/S0033291720003979

8. Gorka SM, Young CB, Klumpp H, et al. Emotion-based brain mechanisms and predictors for SSRI and CBT treatment of anxiety and depression: a randomized trial. *Neuropsychopharmacology*. 2019;44(9):1639-1648. doi:10.1038/s41386-019-0407-7

9. Feurer C, Jimmy J, Bhaumik R, et al. Anterior cingulate cortex activation during attentional control as a transdiagnostic marker of psychotherapy response: a randomized clinical trial. *Neuropsychopharmacol*. 2022;47(7):1350-1357. doi:10.1038/s41386-021-01211-2

10. Schaefer A, Kong R, Gordon EM, et al. Local-Global Parcellation of the Human Cerebral Cortex from Intrinsic Functional Connectivity MRI. *Cerebral Cortex*. 2018;28(9):3095-3114. doi:10.1093/cercor/bhx179

© 2025 Zhang K et al. *JAMA Network Open*.

11. Tian Y, Margulies DS, Breakspear M, Zalesky A. Hierarchical organization of the human subcortex unveiled with functional connectivity gradients. Published online January 14, 2020:2020.01.13.903542. doi:10.1101/2020.01.13.903542

12. King M, Hernandez-Castillo CR, Poldrack RA, Ivry RB, Diedrichsen J. Functional boundaries in the human cerebellum revealed by a multi-domain task battery. *Nat Neurosci*. 2019;22(8):1371-1378. doi:10.1038/s41593-019-0436-x

13. Zhao K, Xie H, Fonzo GA, Carlisle NB, Osorio RS, Zhang Y. Dementia Subtypes Defined Through Neuropsychiatric Symptom–Associated Brain Connectivity Patterns. *JAMA Network Open*. 2024;7(7):e2420479. doi:10.1001/jamanetworkopen.2024.20479

14. Zhao K, Xie H, Fonzo GA, et al. Individualized fMRI connectivity defines signatures of antidepressant and placebo responses in major depression. *Mol Psychiatry*. 2023;28(6):2490-2499. doi:10.1038/s41380-023-01958-8

15. Xia CH, Ma Z, Ciric R, et al. Linked dimensions of psychopathology and connectivity in functional brain networks. *Nat Commun*. 2018;9(1):3003. doi:10.1038/s41467-018-05317-y

16. Wen Z, Pace-Schott EF, Lazar SW, et al. Distributed neural representations of conditioned threat in the human brain. *Nat Commun*. 2024;15(1):2231. doi:10.1038/s41467-024-46508-0

17. Rudie JD, Shehzad Z, Hernandez LM, et al. Reduced Functional Integration and Segregation of Distributed Neural Systems Underlying Social and Emotional Information Processing in Autism Spectrum Disorders. *Cerebral Cortex*. 2012;22(5):1025-1037. doi:10.1093/cercor/bhr171

18. Zhou F, Zhao W, Qi Z, et al. A distributed fMRI-based signature for the subjective experience of fear. *Nat Commun*. 2021;12(1):6643. doi:10.1038/s41467-021-26977-3

© 2025 Zhang K et al. *JAMA Network Open*.
