# Supplementary material for: Functional Connectivity Predicting Transdiagnostic Treatment Outcomes in Internalizing Psychopathologies
Source: JAMA Netw Open. 2025 Sep 3;8(9):e2530008. doi: 10.1001/jamanetworkopen.2025.30008 (PMC12409597; doi:10.1001/jamanetworkopen.2025.30008)
Supplement: Supplement 2. — Data Sharing Statement [file jamanetwopen-e2530008-s002.pdf]

Data Sharing Statement

Zhang. Functional Connectivity Predicting Transdiagnostic Treatment Outcomes in Internalizing Psychopathologies. *JAMA Netw Open*. Published September 03, 2025. doi:10.1001/jamanetworkopen.2025.30008

Data
Data available: Yes
Data types: Deidentified participant data
How to access data: Deidentified participant data and accompanying data dictionary are available at NDA (https://nda.nih.gov/edit_collection.html?id=2134 and https://nda.nih.gov/edit_collection.html?id=2717)
When available: With publication

Supporting Documents
Document types: Statistical/analytic code
How to access documents: Analysis scripts (including the rCCA pipeline and preprocessing code) will be made available upon reasonable request. Requests for access should be directed to: zhenfu.wen@uth.tmc.edu. Alternatively, upon publication, scripts will be deposited to an open-access repository such as GitHub, with updated URLs provided in the final version.
When available: With publication

Additional Information
Who can access the data: Data will be made available to researchers whose proposed use of the data has been approved.
Types of analyses: No restriction.
Mechanisms of data availability: With a signed data access agreement
